# Resveratrol and atherosclerosis: A comprehensive review of its cardioprotective mechanisms and therapeutic potential

**DOI:** 10.21542/gcsp.2025.39

**Published:** 2025-08-30

**Authors:** Razan Moghnieh, Mohammad Kheir Chahine, Riyad Mroweh, Nadim Chaarani, Issa Zalzali, Saleh-Yezan Abdulaal, Amer Yazbak, Hadi Farhat, Joy Raheb Khelo, Razan Abdulaal

**Affiliations:** 1 University of Balamand, Faculty of Medicine and Medical Sciences, Dekwaneh, Beirut, Lebanon; 2 Beirut Arab University, Faculty of Medicine and Medical Sciences, Beirut, Lebanon; 3 Stadtkrankenhaus Korbach, Internal Medicine, Korbach, Germany; 4 Cleveland Clinic, Internal Medicine, Cleveland, Ohio, United States of America

## Abstract

Cardiovascular diseases (CVDs) remain a major global health issue, with atherosclerosis being a primary driver of their development. Atherosclerosis is driven by oxidative stress, chronic inflammation, and lipid dysregulation, which ultimately lead to endothelial dysfunction and plaque formation. Resveratrol, a polyphenolic compound found in grapes and red wine, has emerged as a promising therapeutic agent due to its multivalent protective effects against atherosclerosis. This review outlines the role of resveratrol in the inhibition of key pathologic processes, including suppression of pro-inflammatory signaling pathways, enhancement of antioxidant defense mechanisms, and regulation of cholesterol homeostasis. Resveratrol inhibits NF-κB activation, resulting in decreased vascular inflammation and reduced expression of adhesion molecules such as Vascular Cell Adhesion Molecule-1 (VCAM-1) and Intercellular Cell Adhesion Molecule-1 (ICAM-1), thereby preventing infiltration of immune cells into arterial vessels. Additionally, it increases cholesterol efflux by promoting ATP-binding cassette transporters (ABCA1), enhancing high-density lipoprotein (HDL) function, and lowering lipid levels. Evidence from preclinical and clinical studies suggests that resveratrol holds potential as a natural treatment for the prevention of atherosclerosis.

## Introduction

Atherosclerosis is a progressive inflammatory disease and a leading risk factor for CVDs, which are a major cause of mortality worldwide. It is characterized by lipid accumulation, endothelial dysfunction, and immune cell infiltration within blood vessels^1^. Resveratrol, a natural polyphenol found in grapes and red wine, has been extensively studied for its potential cardiovascular benefits^2^. Emerging research suggests that resveratrol exerts anti-atherosclerotic effects through multiple mechanisms, including anti-inflammatory, antioxidant, and lipid-modulating activities^2^. It has been shown to inhibit the NF-κB signaling pathway, thereby reducing the expression of pro-inflammatory cytokines and adhesion molecules^3^. Furthermore, resveratrol mitigates oxidative stress by enhancing antioxidant enzyme activity and preventing LDL oxidation, which is a critical step in foam cell formation^4^. Additionally, it facilitates cholesterol efflux by upregulating ABCA1, promoting HDL-mediated reverse cholesterol transport^4^. This review focuses on research exploring the anti-atherosclerotic mechanisms of resveratrol. Understanding its molecular and cellular effects is essential for evaluating its role in cardiovascular disease prevention and treatment.

## Methods

This review was conducted in accordance with the Preferred Reporting Items for Systematic Reviews and Meta-Analyses (PRISMA) guidelines. A comprehensive literature search was performed using five major electronic databases: PubMed, Scopus, ScienceDirect, MEDLINE via Ovid, and ClinicalTrials.gov. The search covered all publications from each database’s inception through March 2025. A combination of the following search terms was used: “Resveratrol”, “atherosclerosis”, “cardiovascular disease”, “inflammation”, “cholesterol metabolism”, and “oxidative stress”, applying Boolean operators (AND/OR) as appropriate. No language or geographic restrictions were applied.

The initial search yielded a total of 185 records: 58 from PubMed, 41 from Scopus, 35 from ScienceDirect, 29 from MEDLINE, and 22 from ClinicalTrials.gov. After the removal of 42 duplicate entries, 143 records remained for title and abstract screening. Based on this initial screening, 99 studies were excluded for irrelevance to the topic or lack of mechanistic focus. The full text of 44 articles was retrieved for detailed review. Of these, two could not be accessed due to database or paywall limitations. The remaining 42 articles were assessed for eligibility based on pre-established criteria. Studies were included if they (1) reported experimental or clinical evidence on resveratrol’s effects in the context of atherosclerosis, (2) investigated mechanistic or therapeutic outcomes, and (3) involved in vitro, in vivo, or human subjects relevant to cardiovascular disease. Studies were excluded if they only discussed resveratrol in non-cardiovascular contexts, did not focus on mechanistic pathways, or addressed polyphenol mixtures without isolating resveratrol’s effects.

**Figure 1.**
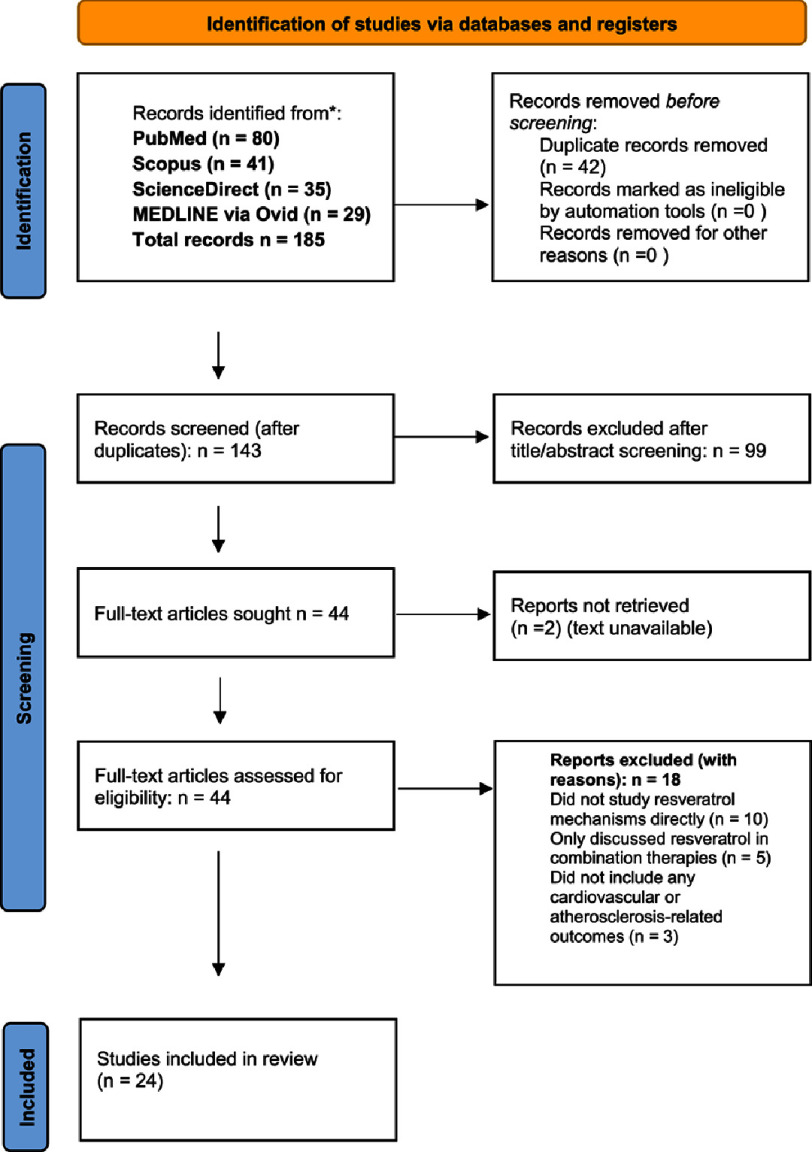
PRISMA flow diagram for the present study.

In total, 24 studies met all inclusion criteria and were included in the final review. The selection process is summarized in the PRISMA flow diagram (Figure 1). The quality of the included studies was assessed using standardized tools: randomized controlled trials were evaluated using the Cochrane Risk of Bias Tool. Study selection and data extraction were performed independently by four reviewers, with disagreements resolved through consensus or consultation with a fifth reviewer.

## Discussion & Results

### Resveratrol-mediated decrease in VCAM, ICAM and interleukins as a mechanism for mitigating atherosclerosis

Resveratrol demonstrates favorable cardiovascular effects, particularly regarding the inhibition of atherosclerosis^5^. Agarwal et al. designed a double-blind, placebo-controlled clinical trial involving 44 healthy subjects. All subjects were randomly allocated to resveratrol supplementation or placebo. The endothelial function and inflammatory biomarkers of the participants were assessed during the study. Resveratrol supplementation was found to be effective in lowering VCAM-1, ICAM-1, and several interleukins^5^. By inhibiting these factors, resveratrol plays a major role in reducing the inflammatory markers that contribute to atherosclerosis and the resulting vascular damage, suggesting that resveratrol may help prevent atherosclerosis.

Based on such evidence regarding resveratrol’s anti-inflammatory and cardiovascular benefits in humans^6^, and using an animal model to further understand the underlying mechanisms, Gyeong-Min et al. described their studies regarding resveratrol’s influence on lipid profiles and atherosclerosis in mice lacking the apolipoprotein E gene (apoE). In this experiment, apoE-deficient mice fed a normal diet received resveratrol supplementation at concentrations of 0.02% and 0.06%, respectively. The mice were observed for an extended period during the study while determining plasma levels of total cholesterol, LDL cholesterol, and HDL cholesterol, as well as hepatic HMG-CoA reductase (HMGR) activity. In this study, hepatic microsomes used for measurement of HMGR and acyl-CoA cholesterol acyltransferase (ACAT) activities were prepared by homogenizing liver tissues and subsequently centrifuging them with radiolabeled substrates.

Histopathological analyses of atherosclerotic lesions removed from the aortic arches were performed after staining, while lipid peroxidation levels were determined using thiobarbituric acid assays and paraoxonase activity levels were assessed using a spectrophotometric method. The atherosclerotic lesions and periarterial fat deposition were also histologically examined. The researchers also assessed the expression of adhesion molecules such as VCAM-1 and ICAM-1 in atherosclerotic vessels. It was found that resveratrol supplementation significantly reduced both total cholesterol and LDL cholesterol levels while increasing HDL cholesterol and the HDL-C/total-C ratio. Moreover, resveratrol supplementation reduced hepatic HMGR activity along with atherosclerotic lesion formation^6^. Thus, it is reasonable to conclude that the anti-atherogenic and hypocholesterolemic properties of resveratrol demonstrate its strong potential.

This broadens our understanding of how resveratrol affects atherosclerosis development and prevention. Cicha et al. in their study emphasize the duality in the distribution of atherosclerotic plaques^7^. They demonstrate that shear stress is one of the critical determinants of plaque localization, which may depend on the interaction of hemodynamic forces and inflammatory cytokines. This study investigates how disturbed flow-induced shear stress promotes endothelial cells to enhance monocyte recruitment and demonstrates the efficacy of simvastatin and resveratrol in mitigating this pro-atherogenic process. The main finding is that laminar shear stress protects endothelial cells against TNF-α-induced upregulation of adhesion molecules and monocyte recruitment through inhibition of TNF signaling pathways.

In contrast, disturbed shear stress increases endothelial sensitivity to TNF-α due to enhanced monocyte adhesion and upregulation of VCAM-1 and E-selectin. Resveratrol is known to be associated with improvement in endothelial function in animal and human studies. Resveratrol inhibits monocyte adhesion to endothelial cells under TNF-α stimulation through interference with NF-κB in response to disturbed shear stress. Furthermore, resveratrol significantly inhibited the TNF-α-mediated increase in VCAM-1 and E-selectin, indicating its potential anti-atherogenic effect by inhibiting leukocyte infiltration in the arterial wall at atherosclerosis-prone sites^7^. This suggests that the anti-inflammatory effects of laminar shear stress can be mimicked by pharmacological compounds like resveratrol that inhibit TNF-α signaling, thus lowering VCAM-1 and ICAM-1 expression.

Complementing such findings about resveratrol and its influence on endothelial function and inflammatory responses, the following study investigated deeper into its therapeutic effects in a model of atherosclerosis and shed light on its vascular protective properties. The anti-inflammatory and anti-atherogenic effects of resveratrol were investigated in a rabbit model of atherosclerosis^8^. Twenty rabbits were divided into two groups: a control group and a resveratrol-treated group, both fed a hypercholesterolemic diet (1% cholesterol) for 56 days. Resveratrol (2 mg/kg/day) was administered to the resveratrol group during the final 28 days. Blood chemistry did not show any significant differences in lipid profiles between groups as measured with an automated analyzer. For histological analysis, atherosclerotic regions were classified with orcein-stained sections, and intimal area and intima/media layer area ratio (IMR) were quantified.

The expression of VCAM-1, MCP-1, and IL-6 was measured using monoclonal antibodies by immunohistochemistry. Results demonstrated marked reductions in atherosclerotic lesions, IMR, and concentrations of VCAM-1, MCP-1, and IL-6 in the resveratrol group, which suggested that resveratrol can restrain the progression of atherosclerosis through anti-inflammatory and vascular protective effects, though no impacts on lipid profiles were observed during this study period. The results indicated that resveratrol significantly diminished the severity of atherosclerotic lesions; thus, in the resveratrol group, only animals with mild lesions were detected, either type I or type II, while 70% of the control group presented with advanced lesions, types III–VI. The IMR was significantly lower in the resveratrol group, indicating less vascular remodeling and intimal hyperplasia. Resveratrol also reduced the expression of VCAM-1, MCP-1, and IL-6, indicating its strong anti-inflammatory properties^8^. However, there was no significant alteration in lipid profiles, which could be attributed to the short duration of the study. These findings have demonstrated that resveratrol decreases the progression of atherosclerosis mainly through reduction of inflammation and vascular injury and thus may be an effective therapeutic agent for cardiovascular disease management.

Based on the findings about the vascular protective effects of resveratrol in atherosclerosis models, Palanisamy et al. further investigated its mechanisms of action, focusing on its anti-inflammatory properties in TNF-α-induced vascular dysfunction^9^. They investigated the impact of resveratrol on endothelial cell responses and systemic vascular inflammation using a well-controlled mouse model and provided additional evidence of its therapeutic potential against cardiovascular diseases. The study reported on the anti-inflammatory impact of resveratrol on TNF-α-induced vascular dysfunction, with an emphasis on its endothelial cell mechanisms in the mouse model. Resveratrol inhibited TNF-α-induced monocyte adhesion to human endothelial cells in a dose-dependent manner at physiologically relevant concentrations (<5 μM), associated with downregulation of mRNA for adhesion molecule ICAM-1 and chemokine monocyte chemoattractant protein-1 (MCP-1).

Mechanistically, resveratrol prevented TNF-α-induced degradation of IκB-α, a critical inhibitor of NF-κB, thereby suppressing nuclear translocation of the NF-κB p65 subunit, which is required for transcription of pro-inflammatory genes. Dietary supplementation of resveratrol in C57BL/6 mice with 0.4% resveratrol in their diet resulted in significant reduction in TNF-α-induced vascular inflammation in vivo. Resveratrol also lowered circulating levels of adhesion molecules sICAM-1, sVCAM-1, and chemokines MCP-1, CXCL1/KC, resulting in inhibition of monocyte adhesion to aortic endothelial cells. Histological examination showed that resveratrol restored elastin fiber integrity in aortic walls and reduced VCAM-1 expression and F4/80-positive macrophage infiltration. These effects were further corroborated by immunohistochemical evidence showing reduced NF-κB activation in aortic tissues of resveratrol-treated mice^9^. The study demonstrates that resveratrol effectively attenuates TNF-α-induced vascular inflammation by inhibiting NF-κB signaling and thus decreasing VCAM-1 expression in mouse aortas.

In addition, resveratrol employs transcriptional regulation of the FERM-kinase for its anti-atherogenic and anti-inflammatory actions^10^. Seo et al. investigated this mechanism using a mouse model of atherosclerosis and cultured endothelial cells. Atherosclerosis was induced by partial carotid ligation in mice, which were treated intraperitoneally with 20 mg/kg of resveratrol daily for 8 days, followed by histological and molecular analyses of aortic tissues. In vitro, resveratrol-treated endothelial cells demonstrated reduced expression of ICAM-1, cleavage of FAK, and monocyte adhesion as assessed by immunoblotting, promoter assays, and adhesion assays, respectively. Histological studies showed that atherosclerotic plaque area was significantly decreased in resveratrol-treated mice, with 50–70% less lipid deposition. In addition, resveratrol treatment diminished mRNA expression of ICAM-1 in the left carotid artery and decreased monocyte adhesion to endothelial cells. At the molecular level, resveratrol increased the expression of lactoferrin, which cleaved FAK into a fragment known as FERM-kinase. This fragment translocated into the nucleus and inhibited ICAM-1 transcription by interacting with Nrf2 at the ARE in the ICAM-1 promoter.

It was shown that lactoferrin-mediated cleavage of FAK and subsequent nuclear localization of the FERM-kinase domain play a critical role in reducing endothelial inflammation. FERM-kinase inhibited TNF-α-induced VCAM-1 expression by inhibiting the transcription factor GATA4, and suppressed monocyte recruitment via downregulation of adhesion molecules such as ICAM-1. Resveratrol exerted its effects through the interaction of FERM-kinase and Nrf2, inhibiting ICAM-1 transcription via the ARE site, with further Nrf2-driven mechanisms downregulating ICAM-1 through NF-κB inactivation. These mechanisms, supported by in vitro results showing a 30–40% reduction in monocyte adhesion within 12 h, highlight the potent anti-inflammatory and anti-atherogenic properties of resveratrol^10^. The study highlights the therapeutic potential of resveratrol against vascular inflammation and atherosclerosis, emphasizing disruption of focal adhesions, enhancement of Nrf2 activity, and regulation of adhesion molecule transcription.

### Resveratrol-mediated decrease in TNF-α/NF-κB signaling pathway decreasing atherosclerosis

In this study conducted by Buttari et al., pretreatment of M1 and M2 macrophage phenotypes with resveratrol significantly reduced TNF-α upregulation in M1 and M2 macrophages exposed to 7-oxocholesterol^11^, which is known to elicit pro-inflammatory responses in macrophages. Additionally, NF-κB is critical in the expression of many inflammatory mediators and chemokines that play a role in atherosclerotic plaque progression. Therefore, by preventing the release of these mediators such as IL-6, IL-12, MCP-1, CCL3, and CCL4, resveratrol not only counteracts the inflammatory status of macrophages but also reduces their ability to induce angiogenesis and plaque instability^11^.

In 2016, Chekalina et al. conducted a randomized controlled clinical trial to study the effect of resveratrol and quercetin on endothelial degeneration factors in CAD patients^12^. The study demonstrated that resveratrol resulted in a significant reduction in TNF-α levels and circulating endothelial microparticles (EMP CD32+CD40+), where both of these markers reflect endothelial dysfunction and inflammation, more effectively than quercetin. Additionally, by inhibiting NF-κB, resveratrol inhibits the inflammatory cascade, which in turn reduces endothelial damage and systemic inflammation that are associated with the progression of coronary artery disease. Furthermore, both resveratrol and quercetin led to a decrease in total cholesterol, which supports their lipid-lowering properties^12^. Thus, the ability of resveratrol to counteract pathogenic processes through protective and anti-inflammatory effects suggests possible therapeutic effects on atherosclerosis and CAD.

Additionally, Froldi and Ragazzi highlighted the effects of resveratrol in inhibiting TNF-α-induced NF-κB activation in coronary endothelial cells^13^. This pathway, as mentioned above, plays a role in inflammation and atherosclerosis progression. By targeting IKK, resveratrol reduced the phosphorylation of NF-κB, which in turn led to a decrease in the production of pro-inflammatory cytokines and adhesion molecules^13^. This anti-inflammatory effect plays a critical role in protecting the vascular endothelium and reducing atherosclerotic plaque progression.

Furthermore, resveratrol has shown that it can effectively reduce TNF-α-induced vascular inflammation by inhibiting the NF-κB signaling pathway. By preventing TNF-α-induced IκB-α degradation, which is a critical step in nuclear translocation of the NF-κB p65 subunit, this attenuates activation of pro-inflammatory genes. Immunofluorescence studies demonstrated that resveratrol significantly decreased nuclear translocation of NF-κB p65 in human endothelial cells, which strengthens the observed anti-inflammatory effects^14^. This in turn disrupts the cascade of expression of adhesion molecules and cytokines such as ICAM-1 and MCP-1, which are important mediators of vascular inflammation. This suggests that resveratrol, when used at physiologically relevant levels, can be used as a therapeutic agent for vascular endothelial dysfunction^14^.

As demonstrated by Cicha et al., similar to resveratrol, physiologic laminar shear stress inhibits the association of TNFR-1 with TRAF-2, which blocks TNF-α receptor activation and thus prevents activation of the inflammatory cascade including NF-κB^15^. On the other hand, non-uniform shear stress, which occurs typically at arterial bifurcations in the body, aggravates TNF-α-induced endothelial activation, leading to increased recruitment and adhesion of monocytes. This is mediated by increasing the expression of adhesion molecules such as VCAM-1 and E-selectin. Resveratrol reduced monocyte recruitment and endothelial inflammation up to 50% by counteracting shear stress effects through suppressing NF-κB-mediated upregulation of VCAM-1 and E-selectin^15^. These findings highlight the therapeutic potential of resveratrol in modulating the TNF-αNF-κB signaling pathway, which plays a major role in the pathogenesis and progression of atherosclerotic plaques. A summary of selected clinical trials evaluating resveratrol’s cardiovascular effects in human subjects is presented in Table 1.

**Table 1:** Summary of selected clinical trials on resveratrol in cardiovascular health.

**Study**	**Population**	**Dose / Duration**	**Outcomes**	**Findings**
Chekalina et al., 2016	CAD patients (*n* = 60)	40 mg/day + Quercetin, 60 days	TNF-α, EMPs	↓Inflammatory markers, better than Quercetin
Agarwal et al., 2013	Healthy adults (*n* = 44)	250 mg/day, 30 days	VCAM-1, ICAM-1	↓Adhesion molecules, improved endothelial markers
Timmers et al., 2011	Obese men (*n* = 11)	150 mg/day, 30 days	Metabolic markers	Improved insulin sensitivity, but no lipid change
Wong et al., 2011	Type 2 diabetes (*n* = 19)	500 mg/day, 4 weeks	Endothelial function	No significant effect

### Resveratrol-mediated decrease in LPL expression in macrophages preventing atherosclerosis

Low-dose resveratrol appeared to have significant therapeutic potential for reducing atherosclerosis by altering macrophage functions^16^. As demonstrated by Azorín-Ortuñoa et al., resveratrol specifically counteracts the pro-atherogenic effects of high-fat diet by downregulating fatty acid-binding protein 4 (FABP4) and lipoprotein lipase (LPL) expression in macrophages. This effect, which was observed in pretreated peripheral blood mononuclear cells (PBMCs) and macrophages with oxidized LDL, sheds light on an important pathway in lipid accumulation and inflammation that is disrupted by resveratrol. The decrease in FABP4 expression, which plays a significant role in the inflammatory and metabolic processes that lead to atherosclerotic plaque formation, is notable^16^. All these findings underscore the significant dietary role that resveratrol may play in the management and prevention of cardiovascular diseases by targeting the early molecular events that occur during atherogenesis.

As mentioned by Berrougui et al., resveratrol has shown significant atheroprotective effects through alteration of macrophage lipid metabolism^17^. It downregulates the expression of LPL and scavenger receptor AII (SR-AII) in macrophages, which reduces lipid uptake by these cells. Specifically, decreased activity of LPL reduces lipid uptake, while reduced SR-AII expression on the surface of macrophages inhibits absorption of oxidized LDL. All of these effects inhibit foam cell formation, which is a key mediator in the formation and progression of atherosclerotic plaques^17^. Moreover, resveratrol’s suppression of macrophage-mediated oxLDL uptake leads to reduction of inflammation and oxidative stress, both of which are important drivers of atherosclerosis.

### Resveratrol-mediated decrease in LDL oxidation at atherosclerotic sites

A controlled laboratory study conducted in 2009 by Berrougui et al. investigated resveratrol’s role as an atheroprotective compound by examining its effects on lipid peroxidation and oxidative stress^18^. For this study, human plasma was collected to isolate LDL and HDL, allowing researchers to assess resveratrol’s antioxidant effects on LDL peroxidation. LDL oxidation was induced using copper ions or exposure to oxygen free radicals, and the formation of conjugated dienes was measured to evaluate oxidation levels. Results showed that resveratrol, in a concentration-dependent manner, inhibited the rise in conjugated dienes caused by CuSO_4_ and gamma-radiolysis, extending the lag phase and reducing the maximum oxidation rate (Vmax). This suggests that resveratrol acts as a chelator, with vitamin E serving as a positive control, as resveratrol slowed its degradation. Additionally, the study explored HDL’s role in promoting reverse cholesterol transport (RCT) by enabling the efflux of free cholesterol from macrophages. HDL oxidation was studied using CuSO_4_ to stimulate oxidative stress, and resveratrol was shown to decrease conjugated dienes (oxidative markers), preserving HDL’s ability to mediate cholesterol efflux^18^. These findings revealed that resveratrol has natural antioxidant effects and the ability to promote cholesterol efflux, thus potentially preventing and treating cardiovascular diseases.

Another controlled in vitro study by Brito et al. (2008) examined the inhibitory effects of resveratrol on another signaling pathway, showing its anti-atherogenic properties^19^. Oxidized LDL, a key contributor to atherosclerosis, promotes mTOR pathway activation to regulate cellular processes and proliferate smooth muscle cells (SMCs). This occurs through the PI3K/Akt signaling cascade and the sphingolipid pathway. Resveratrol, with its antioxidant properties, inhibits the PI3K/Akt signaling cascade, which is necessary for activation of the mTOR pathway^19^. Thus, it prevents oxLDL-induced SMC proliferation.

Rapamycin, a specific inhibitor of mTOR, is used to assess the dependence of oxLDL on mTOR for inducing DNA synthesis and SMC proliferation. Additionally, wortmannin and LY294002, PI3K inhibitors, are used to block the phosphorylation of PDK1, Akt, and mTOR, preventing SMC proliferation. Further studies have explored additional pathways, such as metalloproteases and sphingolipid mediators, which promote oxLDL-induced SMC proliferation independently of the PI3K/Akt/mTOR signaling pathway. Small doses of resveratrol inhibit ERK1/2 phosphorylation, which plays a role in DNA synthesis. While AMPK is activated by resveratrol to inhibit the mTOR pathway, it does not affect the presence of oxLDL. Therefore, it is not involved in the inhibitory effect of resveratrol on the PI3K/Akt/mTOR pathway activated by oxLDL. The effect of resveratrol is concentration-dependent for its apoptotic effect, inhibiting pathways to reduce SMC and cardiac fibroblast proliferation.

Turning our attention to a different study by Deng et al. (2015), which focused on the functions and mechanisms of resveratrol on NLRP3 inflammasomes during vascular injury^20^. For this, they used male Sprague-Dawley rats on a high-cholesterol diet, giving them vitamin D2 to aggravate vascular injury. For the experiment, the rats were divided into four groups: normal control (NC), RSV-treated group (RSV), hypercholesterolemia and vitamin D2 group (HC+VD), and the RSV-treated hypercholesterolemia and vitamin D2 group (RSV+HC+VD). Researchers concluded that RSV reduced lipid levels, total cholesterol, LDL, and triglyceride (TG) levels, but increased HDL levels. Thus, reducing both cholesterol crystal formation and the expression of LOX-1, which is a receptor for oxLDL. Moreover, RSV regulated oxidative stress by enhancing superoxide dismutase (SOD) and glutathione peroxidase (GPx) activities while decreasing malondialdehyde (MDA) levels. Additionally, RSV reduced inflammation by regulating the NF-κB and p38 MAPK pathways, which suppressed NLRP3 inflammasome transcription^20^. This study concluded that hypercholesterolemia-associated vascular disorders can be treated with resveratrol by inhibiting inflammasome activation.

A study conducted by Rocha et al. (2009) demonstrated that resveratrol may have beneficial effects in high-fat diets where it decreased oxLDL, serum, and hepatic oxidative stress^21^. The experiment in this study compared the effect of resveratrol in high-fat diet and normal-fat diet conditions. For this, 24 male Wistar rats were used and divided into four groups: control group fed standard diet without resveratrol, a standard diet group with resveratrol (6 mg/L in drinking water), a high-fat diet-fed group without resveratrol, and a high-fat diet group with resveratrol. In high-fat diet rats, the levels of glucose, LDL cholesterol, and hepatic lipid hydroperoxide were elevated, causing hyperglycemia, dyslipidemia, and heightened oxidative stress in the liver. After administering resveratrol to this group, oxidized LDL and glucose levels were reduced. Additionally, SOD activity was enhanced, reducing reactive oxygen species so that hepatic oxidative stress was reduced. Compared to standard diet rats, giving resveratrol to high-fat diet animals reduced oxidative stress by decreasing the levels of hepatic hydroperoxide and oxidized glutathione levels^21^. In conclusion, resveratrol works in high-fat diet conditions as an antioxidant by increasing superoxide dismutase to reduce oxLDL and hepatic oxidative stress.

Moving to the study by Voloshyna et al. (2013), which examined resveratrol’s anti-atherogenic effects by reducing foam cell formation and oxLDL uptake^22^. Researchers induced foam cell formation by adding acetylated LDL to THP-1 macrophages, then treated these macrophages with resveratrol (10 μM or 25 μM). They then assessed lipid accumulation in these macrophages by staining them with Oil Red O dye that binds lipids and visualized them under light microscopy. Compared to untreated controls, findings showed that resveratrol reduced foam cell formation by 40.26%. This demonstrated the role of resveratrol in suppressing lipid accumulation in macrophages. Additionally, to monitor whether resveratrol impacts oxLDL uptake in macrophages, they fluorescently labeled them with oxLDL in the absence and presence of resveratrol. Using a confocal microscopy system, researchers measured the fluorescence intensity to indicate oxLDL uptake, and it appeared that resveratrol reduced LDL uptake by 28.9%^22^. Thus, resveratrol inhibits macrophage uptake of oxidized lipoproteins and foam cell formation, which is a critical step in atherosclerosis.

### Resveratrol modulates ApoA1, ABCA1, and HDL: implications for cholesterol metabolism and atherosclerosis

The controlled laboratory study conducted in 2009 by Berrougui et al. also demonstrated that resveratrol enhances cholesterol efflux from macrophages by upregulating ABCA1 expression, promoting apoA-I binding to ABCA1, and stimulating HDL activity, ultimately reducing atherosclerosis^23^. First, J774 cell line macrophages were loaded with ^3^H-cholesterol and treated with different concentrations of resveratrol (0–25 μM) and compared with a cAMP-positive control. Researchers measured apoA-I-mediated cholesterol efflux by calculating the percentage of radiolabeled cholesterol released from the cells into the medium. This showed that resveratrol increased apoA-I-mediated cholesterol efflux in a dose-dependent manner (*R*^2^ = 0.907, *p* < 0.05) and enhanced the expression of ABCA1 in macrophages, promoting cholesterol efflux. Second, cholesterol influx was studied in J774 macrophages using radiolabeled cholesterol. Resveratrol reduced cholesterol influx into macrophages in a concentration-dependent manner (*R*^2^ = 0.89, *p* < 0.05), which was linked to downregulation of lipid uptake genes such as LPL and SR-AII. Finally, researchers demonstrated that resveratrol’s ability to preserve HDL’s functionality under oxidative conditions is critical for maintaining effective reverse cholesterol transport (RCT)^23^. By enhancing ABCA1 expression, promoting apoA-I binding, and mitigating oxidative stress, resveratrol supports the prevention and treatment of atherosclerosis through its multifaceted role in cholesterol metabolism and transport.

The study by Voloshyna et al. (2013) not only showed the role of resveratrol in reducing foam cell formation and oxLDL uptake, but also assessed cholesterol efflux from macrophages through different pathways via the PPAR-γ and adenosine 2A receptor pathways^24^. The experiment utilized two types of cell cultures: THP-1 monocytes/macrophages and human arterial endothelial cells (HAECs). As mentioned previously, resveratrol was added at varying concentrations, and its effects on foam cell formation and cholesterol metabolism were monitored. OxLDL was used to induce foam cell formation, and fluorescently labeled oxLDL allowed for detailed monitoring of macrophage uptake. ApoA-I and HDL were used for cholesterol efflux assays.

In addition to the primary findings, the study also demonstrated that resveratrol enhanced apoA-I-mediated cholesterol efflux by 21.6% and HDL-mediated efflux by over 2-fold, indicating its role in promoting reverse cholesterol transport^24^. The expression of key cholesterol efflux proteins was upregulated by resveratrol, including ABCA1 (by 68–168%), ABCG1 (by 69%), and CYP27A1 (by 65–81%). These findings highlight resveratrol’s pivotal role in enhancing cholesterol efflux and promoting cholesterol metabolism. To verify the pathways involved, PPAR-γ expression was analyzed, and its role was confirmed by siRNA silencing. The silencing blocked the resveratrol-induced upregulation of cholesterol efflux proteins such as ABCA1, ABCG1, and SR-BI. Additionally, 27-hydroxylase expression was significantly increased, highlighting enhanced cholesterol metabolism and oxysterol elimination. Furthermore, resveratrol activated adenosine 2A receptors, promoting cholesterol efflux, as confirmed using A2A receptor antagonists. Notably, the upregulation of ABCA1 and CYP27A1 by resveratrol was blocked when PPAR-γ was inhibited, showing that these effects are PPAR-γ-dependent. Similarly, adenosine receptor blockade abolished the beneficial effects of resveratrol on cholesterol efflux, implicating adenosine signaling as a key mediator.

Under controlled experimental conditions (37 °C, 5% CO_2_), HAECs, THP-1, and peripheral blood mononuclear cells (PBMCs) were cultured with specific growth media^24^. Resveratrol, at concentrations of 10 μM and 25 μM, was applied. Cells treated with the PPAR-γ antagonist (GW9662) or A2A receptor antagonist (ZM-241385) revealed the necessity of these pathways for resveratrol’s effects. Gene expression analysis confirmed increased mRNA levels of ABCA1, ABCG1, and 27-hydroxylase, further supporting the conclusion that resveratrol facilitates cholesterol efflux and reduces foam cell formation^24^.

Additional clinical evidence has shown mixed outcomes regarding resveratrol supplementation in human subjects. Timmers et al. conducted a randomized controlled trial involving obese but otherwise healthy men, where resveratrol (150 mg/day for 30 days) improved insulin sensitivity and mitochondrial function but did not significantly affect lipid profiles or inflammatory markers. This highlights that resveratrol’s metabolic benefits may not uniformly extend to cardiovascular endpoints. Similarly, Wong et al. examined the effects of high-dose resveratrol (500 mg/day for four weeks) in patients with type 2 diabetes and found no significant improvement in endothelial function or glycemic control. These findings reinforce the need for larger, longer-duration trials to clarify resveratrol’s therapeutic role in clinical cardiovascular settings.

Despite a wide array of studies supporting resveratrol’s anti-atherogenic mechanisms, the literature also presents conflicting results. For instance, while Agarwal et al. reported reductions in VCAM-1 and ICAM-1 in healthy adults, Timmers et al. found that resveratrol supplementation in obese men improved insulin sensitivity without significantly affecting lipid profiles or inflammatory markers^5, 6^. Similarly, Chekalina et al. observed decreased TNF-α levels and endothelial microparticles in CAD patients, yet other trials with similar doses failed to replicate these findings consistently^12^. Variability in dosing regimens, formulations, duration, and study populations likely contributes to these discrepancies. Additionally, small sample sizes and lack of long-term follow-up in many clinical studies limit the generalizability of results. These inconsistencies highlight the need for larger, well-controlled trials to validate resveratrol’s efficacy in cardiovascular disease prevention.

While the protective effects of resveratrol have been widely demonstrated in preclinical studies, translating these findings into consistent clinical outcomes remains a challenge due to the compound’s poor bioavailability. Resveratrol is rapidly metabolized in the intestine and liver, resulting in low systemic concentrations of the active compound^13^. This may explain why some clinical trials, such as those by Timmers et al. and Wong et al., reported modest or no significant effects on lipid profiles and inflammatory markers despite using pharmacologically relevant doses^6, 25^. Even studies that demonstrated improvements in endothelial function or reductions in biomarkers, like those by Agarwal et al. and Chekalina et al., often used short durations and small sample sizes^5, 12^. Various strategies have been explored to overcome these limitations, including the development of resveratrol analogs (e.g., pterostilbene), nanoformulations, and co-administration with bioenhancers like piperine, but these approaches require further clinical validation. Therefore, while resveratrol remains a promising nutraceutical, its clinical application depends heavily on overcoming its pharmacokinetic barriers.

## Conclusion

Resveratrol has emerged as a promising compound for managing atherosclerosis, which remains a major health concern. It exerts its protective effects through several mechanisms, including reducing the expression of VCAM-1, ICAM-1, and interleukins, which are key players in inflammation. Additionally, resveratrol mitigates atherosclerosis by decreasing the TNF-α/NF-κB signaling pathway and reducing LPL expression in macrophages. Furthermore, resveratrol influences cholesterol metabolism by modulating apoA-I, ABCA1, and HDL levels. While the therapeutic potential of resveratrol is evident, challenges such as bioavailability, optimal dosage, and long-term safety still need to be addressed through further research and human trials.

From a clinical perspective, resveratrol should not yet be considered a standalone treatment for atherosclerosis but rather a potential adjunct in comprehensive cardiovascular prevention strategies. Given its favorable safety profile and anti-inflammatory properties, resveratrol supplementation may be appropriate for select patients, particularly those with metabolic syndrome or early-stage atherosclerotic disease, pending further evidence. Clinicians should be aware of its pharmacokinetic limitations and avoid overreliance on unstandardized supplements, especially when evidence of benefit in large-scale human trials remains limited. Until more robust clinical guidelines are developed, resveratrol may be best positioned as a complementary therapy, ideally discussed in the context of lifestyle interventions and shared decision-making with patients.
